# Divergence-free reconstruction for accelerated 3D phase-contrast flow measurements

**DOI:** 10.1186/1532-429X-14-S1-W38

**Published:** 2012-02-01

**Authors:** Julia Busch, Daniel Giese, Lukas Wissmann, Sebastian Kozerke

**Affiliations:** 1 Institute for Biomedical Engineering, University and ETH Zurich, Zurich, Switzerland; 2 Division of Imaging Sciences and Biomedical Engineering, King's College London, London, UK

## Summary

In this work an approach is described to efficiently reduce inaccuracies of flow data acquired with reduced data acquisition methods based on the physical prior knowledge of zero divergence in 3D velocity vector fields. The divergence-free condition is implemented using a synergistic combination of normalized convolution and divergence-free radial basis functions. The efficacy of the method is demonstrated for aortic flow measurements obtained from undersampled data.

## Background

Cine 3D phase-contrast magnetic resonance imaging (PC-MRI) has emerged as a valuable tool for assessing blood flow patterns [Markl, JCMR’11]. The relatively long acquisition times, however, limit its application in a clinical setting. Over the last years several undersampling techniques such as TSENSE, GRAPPA, PEAK-GRAPPA, k-t SENSE, k-t PCA and Compressed Sensing have been introduced which allow for up to 6-fold acceleration in typical applications [Kozerke, JCMR’08]. Reduced data acquisition schemes may, however, induce additional artifacts, correlated noise and temporal smoothing and hence may compromise overall confidence in the flow data [Blaimer, MRM’11]. It is the objective of the present work to correct for parts of the error by taking into account the physical prior of zero divergence into flow field reconstruction.

## Methods

Normalized convolution [Knutsson, CVPR’93] describes a local expansion of image data into basis functions by convolution. Divergence-free basis functions [Lowitzsch, Texas’02] were used in combination with normalized convolution in order to incorporate prior knowledge of blood incompressibility into MR image reconstruction of fully sampled as well as undersampled phase contrast images.

In-vivo cine 3D PC-MRI data of the aorta was acquired in 5 healthy volunteers using a 6-element cardiac coil array on a 3T Philips Achieva systems (Philips Healthcare, Best, The Netherlands). 24 heart phases and 26-34 slices were recorded at a spatial resolution of 1.43mm x 1.43mm x 1.75mm. 2x, 4x and 8x undersampling with 75% partial Fourier sampling was simulated. Images were reconstructed with TSENSE (2x,4x) and with k-t PCA (2x,8x). For k-t PCA reconstruction 55 training profiles were used.

## Results

**Figure 1:**
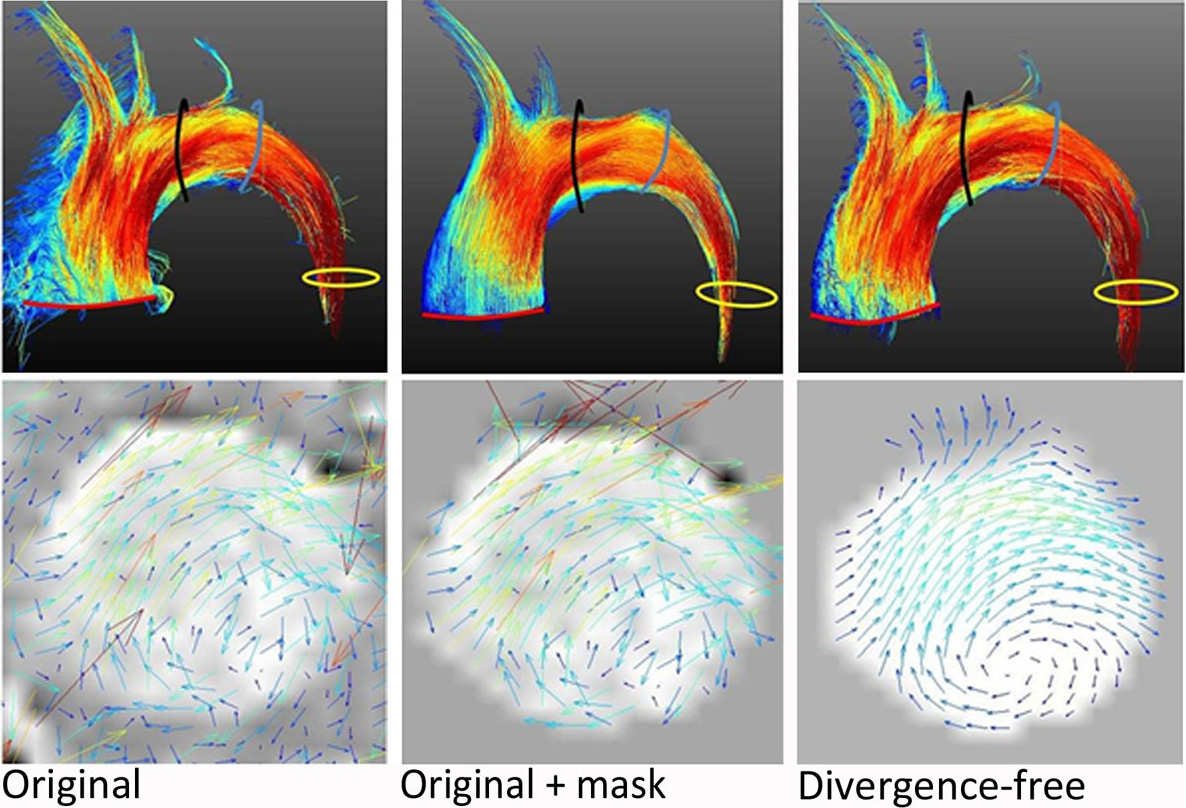
Streamline visualization and illustration of in-plane velocities at the position of the black contour in the aorta in the original data, magnitude-based masked data and data after divergence-free reconstruction.

Figure 1 illustrates flow through the aortic arch from a fully sampled data set, the masked data set and the data set reconstructed with the proposed method. Streamline visualization indicates a reduction in noise and outflow upon divergence-free reconstruction.

**Figure 2:**
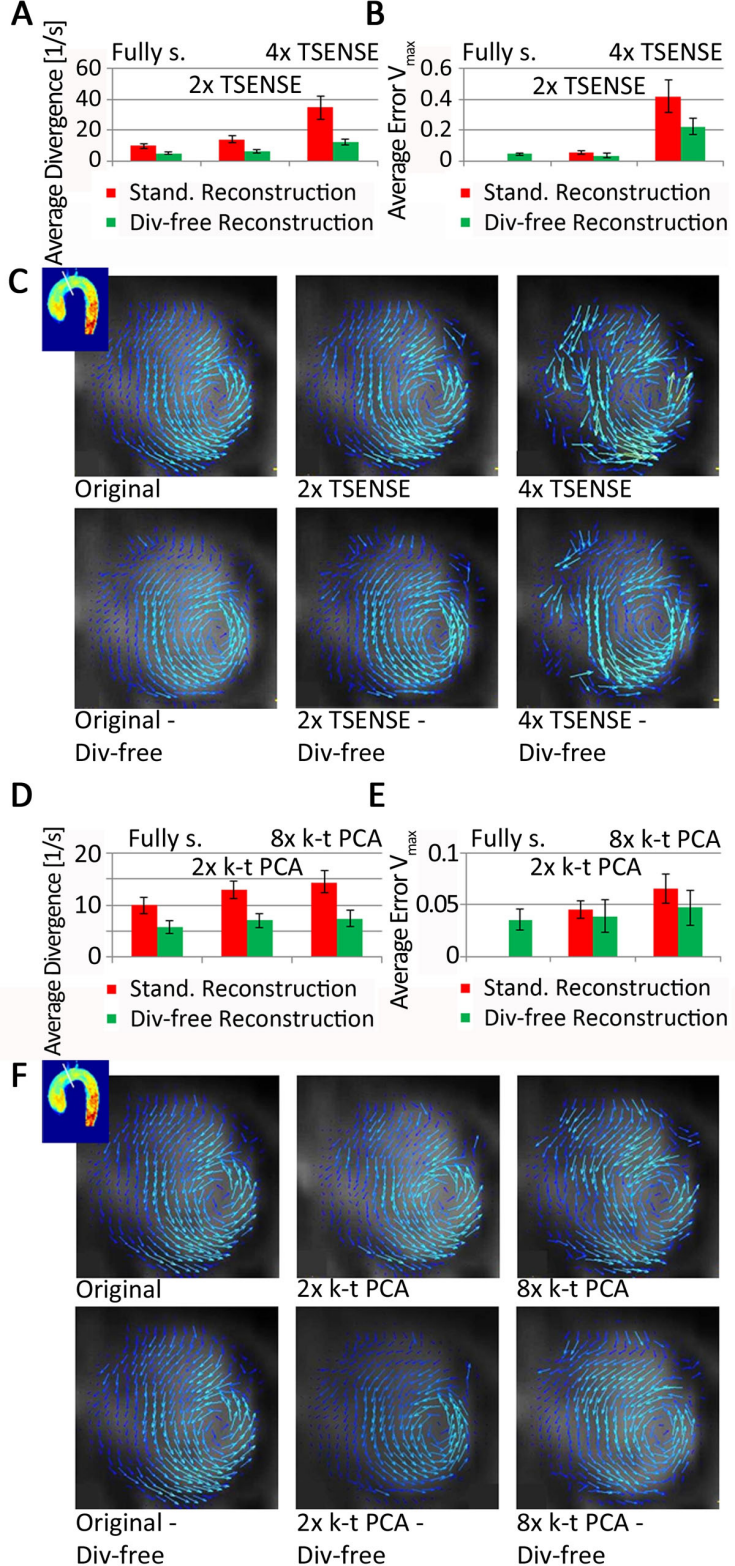
Average divergence over the aorta of 5 healthy volunteers for standard and divergence-free reconstruction of fully sampled, 2x and 4x TSENSE data (A) and fully sampled, 2x and 8x k-t PCA data (D). Average error in maximum velocity for standard and divergence-free reconstruction of fully sampled, 2x and 4x TSENSE data (B) and fully sampled, 2x and 8x k-t PCA data (E). In-plane flow pattern in the ascending aorta of one volunteer for fully sampled, 2x and 4x TSENSE (C) and fully sampled, 2x and 8x k-t PCA (F) before and after divergence-free reconstruction.

Furthermore, divergence-free reconstruction leads to a reduction in errors in flow direction, which can be seen in improved in-plane flow pattern. In Figure 2A and B average divergence and average deviation in maximum velocity is compared for fully sampled, 2x and 4x TSENSE in-vivo data. Divergence-free reconstruction reduces the divergence by 50±2%, 57±2% and 64±3%, respectively and the error in maximum velocity decreased by 35±12% and 47±4%, respectively. The visualization of in-plane flow pattern shows clearer structures (Fig 2C). For 2x and 8x k-t PCA similar results are seen (Fig 2D-F).

## Conclusions

It has been shown that image quality of 3D flow measurements reconstructed from undersampled PC-MRI data can be greatly improved by the use of divergence-free basis functions. Hence, this approach shows considerable potential to compensate for increased noise and local data inconsistencies associated with undersampling strategies employed for reducing the long scan times associated with cine 3D PC-MRI.

